# EuCARE-hospitalised study protocol: a cohort study of patients hospitalised with COVID-19 in the EuCARE project

**DOI:** 10.1186/s12879-023-08658-2

**Published:** 2023-10-16

**Authors:** Pontus Hedberg, Benedetta Varisco, Francesca Bai, Anders Sönnerborg, Pontus Naucler, Nico Pfeifer, Alessandro Cozzi-Lepri, Francesca Ceccherini-Silberstein, Daniel Naumovas, Francis Drobniewski, Björn-Erik Ole Jensen, Cristina Toscano, Miłosz Parczewski, Gibran Horemheb Rubio Quintanares, Matilu Mwau, Jorge A. Pinto, Francesca Incardona, Chiara Mommo, Giulia Marchetti

**Affiliations:** 1 Division of Infectious Diseases, Department of Medicine Huddinge, Karolinska Institutet, Stockholm, Sweden; 2 Department of Health Sciences, University of Milan, Clinic of Infectious Diseases, San Paolo Hospital, ASST Santi Paolo E Carlo, Milan, Italy; 3 Department of Infectious Diseases, Karolinska University Hospital, Stockholm, Sweden; 4 Institute for Bioinformatics and Medical Informatics, University of Tübingen, Tübingen, Germany; 5 Centre for Clinical Research, Epidemiology, Modelling and Evaluation (CREME), Institute for Global Health, UCL, London, UK; 6 Department of Experimental Medicine, University of Rome Tor Vergata, Rome, Italy; 7 Vilnius Santaros Klinikos Biobank, Vilnius University Hospital Santaros Klinikos, Vilnius, Lithuania; 8 Department of Infectious Disease, Imperial College London, London, UK; 9 Department of Medical Microbiology, University Hospitals Dorset, Poole, UK; 10 Department of Gastroenterology, Hepatology and Infectious Diseases, Medical Facultyand, University Hospital Düsseldorf, Heinrich Heine University, Düsseldorf, Germany; 11 Microbiology Laboratory, Centro Hospitalar de Lisboa Ocidental, Lisbon, Portugal; 12 Department of Infectious, Tropical Diseases and Immune Deficiency, Pomeranian Medical Universityin, Szczecin, Szczecin, Poland; 13 Infectious Diseases Department, Instituto Nacional de Ciencias Médicas y Nutrición Salvador Zubirán, Mexico City, Mexico; 14 Paul Ehrlich Institut, Virus Safety, Virology Department, Langen, Germany; 15 Center for Infectious and Parasitic Diseases Control Research, Kenya Medical Research Institute, Busia, Kenya; 16 Department of Pediatrics, Pediatric Immunology Division, School of Medicine, Universidade Federal de Minas Gerais (UFMG), Belo Horizonte, MG Brazil; 17 IPRO-InformaPRO S.R.L., Rome, Italy; 18 EuResist Network GEIE, Rome, Italy

**Keywords:** Hospitalised COVID-19 patients; COVID-19 mortality; SARS-CoV-2 variants; SARS-CoV-2 vaccination and immunity

## Abstract

**Background:**

Severe acute respiratory syndrome coronavirus 2 (SARS CoV-2), the virus responsible for coronavirus disease 2019 (COVID-19), can lead to hospitalisation, particularly in elderly, immunocompromised, and non-vaccinated or partially vaccinated individuals. Although vaccination provides protection, the duration of this protection wanes over time. Additional doses can restore immunity, but the influence of viral variants, specific sequences, and vaccine-induced immune responses on disease severity remains unclear. Moreover, the efficacy of therapeutic interventions during hospitalisation requires further investigation. The study aims to analyse the clinical course of COVID-19 in hospitalised patients, taking into account SARS-CoV-2 variants, viral sequences, and the impact of different vaccines. The primary outcome is all-cause in-hospital mortality, while secondary outcomes include admission to intensive care unit and length of stay, duration of hospitalisation, and the level of respiratory support required.

**Methods:**

This ongoing multicentre study observes hospitalised adult patients with confirmed SARS-CoV-2 infection, utilising a combination of retrospective and prospective data collection. It aims to gather clinical and laboratory variables from around 35,000 patients, with potential for a larger sample size. Data analysis will involve biostatistical and machine-learning techniques. Selected patients will provide biological material. The study started on October 14, 2021 and is scheduled to end on October 13, 2026.

**Discussion:**

The analysis of a large sample of retrospective and prospective data about the acute phase of SARS CoV-2 infection in hospitalised patients, viral variants and vaccination in several European and non-European countries will help us to better understand risk factors for disease severity and the interplay between SARS CoV-2 variants, immune responses and vaccine efficacy. The main strengths of this study are the large sample size, the long study duration covering different waves of COVID-19 and the collection of biological samples that allows future research.

**Trial registration:**

The trial has been registered on ClinicalTrials.gov. The unique identifier assigned to this trial is NCT05463380.

## Background

Since the onset of the coronavirus disease 2019 (COVID-19) pandemic, novel variants of severe acute respiratory syndrome coronavirus 2 (SARS-CoV-2) have emerged. The World Health Organization (WHO) categorises these variants based on level of concern, including variant of interest (VOI), variant of concern (VOC), and variants under monitoring (VUM) [1]. The VOC known as Omicron (B.1.1.529) was designated by the WHO on November 26, 2021 [2]. In contrast, the European Centre for Disease Prevention and Control (ECDC) removed BA.2, BA.4, and BA.5 variants from their list of concern due to their limited circulation [3].

To better understand the impact of these variants, detailed information is needed regarding their severity, mortality rates, disease progression, and the effectiveness of currently available vaccines and therapeutic interventions. Notably, previous reports have suggested increased disease severity for the B1.1.1.7 (alpha) variant compared to other co-existing and previous variants [4–6].

Recent studies have indicated that the Omicron variant poses lower risks of hospital attendance, admission, and death when compared to the Delta variant [7–9]. While the likelihood of severe disease from Omicron is lower compared to previous variants, the sheer volume of cases associated with it has led to a cumulative increase in hospitalisations related to COVID-19, surpassing those caused by other variants [10]. The diminished likelihood of severe disease associated with Omicron could be a result of partial immunity acquired through previous infection or vaccination. Additionally, findings from animal studies, wherein Omicron infections exhibited lower viral levels in infected tissues, lend further support to the notion that Omicron may inherently induce a less severe infection when compared to alternative variants [11].

The influence of different SARS-CoV-2 variants on COVID-19-specific therapies, such as antivirals and immunomodulators, remains to be thoroughly elucidated. Evidence suggests that widely used monoclonal antibody cocktails exhibit reduced efficacy against the Omicron variant, while antivirals continue to be effective [12–17].

The complexity of the situation is further compounded by multiple factors, including immunity derived from prior infections and COVID-19 vaccination. Although the impact of virus variants is presently focused largely on the spike protein, the potential influence of mutations in other regions of the SARS-CoV-2 genome on clinical outcomes and vaccine response necessitates further investigation. Advanced machine-learning techniques can facilitate the analysis of complex relationships between variables in this context.

Thus, our current understanding of the interplay between SARS-CoV-2 variants, disease severity, vaccine efficacy, treatment effectiveness, and immune responses is insufficient, and requires more research. The European Cohorts of Patients and Schools to Advance Response to Epidemics (EuCARE) project, with its access to diverse data from multiple European and non-European countries, including viral sequences, holds significant value.

## Methods and design

### The EuCARE project

The EuCARE project is a collaborative effort between several cohorts of patients and schools across various geographic regions, including European and non-European countries (www.eucareresearch.eu). The project aims to provide an advanced response to COVID-19 epidemics by consolidating or expanding interactions among different clinical centres and research centres. The project brings together a comprehensive, multidisciplinary team of clinicians, virologists, epidemiologists, statisticians, and experts in artificial intelligence. The project aims to investigate several aspects of the COVID-19 pandemic, including natural and vaccine-induced immunity to different viral variants in healthcare workers, the clinical course and long-term follow-up of hospitalised COVID-19 patients, and the best strategies to control the spread of different viral variants in schools [18]. The study focuses on different groups, including hospitalised patients, patients with post-acute sequelae of SARS-CoV-2 infection (PASC), vaccinated healthcare workers (HCW), and school cohorts.

As part of the EuCARE project, Work Package 3 (WP3) has been designed to investigate the impact of COVID-19 on both hospitalised and outpatient populations. WP3 includes two distinct cohorts: the EuCARE Hospitalised Study, which oversees collecting data from COVID-19 patients during the acute phase of hospitalisation, and the EuCARE POSTCOVID Study, which gathers data from both hospitalised and outpatient COVID-19 patients. The focus of this paper is the EuCARE Hospitalised Study, and we present its protocol here.

### Aims and objectives

The aims of this study are to analyse the clinical course of COVID-19 in hospitalised patients in relation to SARS-CoV-2 viral characteristics and vaccines used and to provide recommendations for optimised clinical management and treatment, taking into consideration the viral characteristics and vaccines employed. Data and biological material from COVID-19 inpatients in a diverse setting of twelve hospitals/clinics across eleven countries and four continents are being collected and will be analysed.

The specific objectives of this study are:

- To describe the patterns of clinical symptoms, therapeutic interventions, and clinical outcomes in patients hospitalised for SARS CoV-2 infection.
- To assess the impact of viral variants/sequences on clinical outcomes measured by all-cause in-hospital mortality, non-invasive mechanical ventilation/high-flow nasal oxygen, mechanical ventilation, admission to the intensive care unit (ICU), length of ICU stay, and overall length of hospitalisation.
- To assess the impact of different vaccines and vaccination schedules on clinical outcomes (vaccine effectiveness for progression/pathogenesis) measured by all-cause in-hospital mortality, non-invasive mechanical ventilation/high-flow nasal oxygen, mechanical ventilation, admission to the ICU, length of ICU stay, and overall length of hospitalisation, with the goal of enhancing our comprehension of vaccine hesitancy.
- To assess the impact of viral variants/sequences, different vaccines and vaccination schedule on response to treatment interventions and the associated clinical outcomes.
- To provide biological material to Work Package 2 (WP2) in EuCARE for further studies on viral characteristics and associated immune responses.
- To provide data to Work Package 5 (WP5) in EuCARE for further studies using machine-learning approaches.

### Study design

**Fig. 1:**
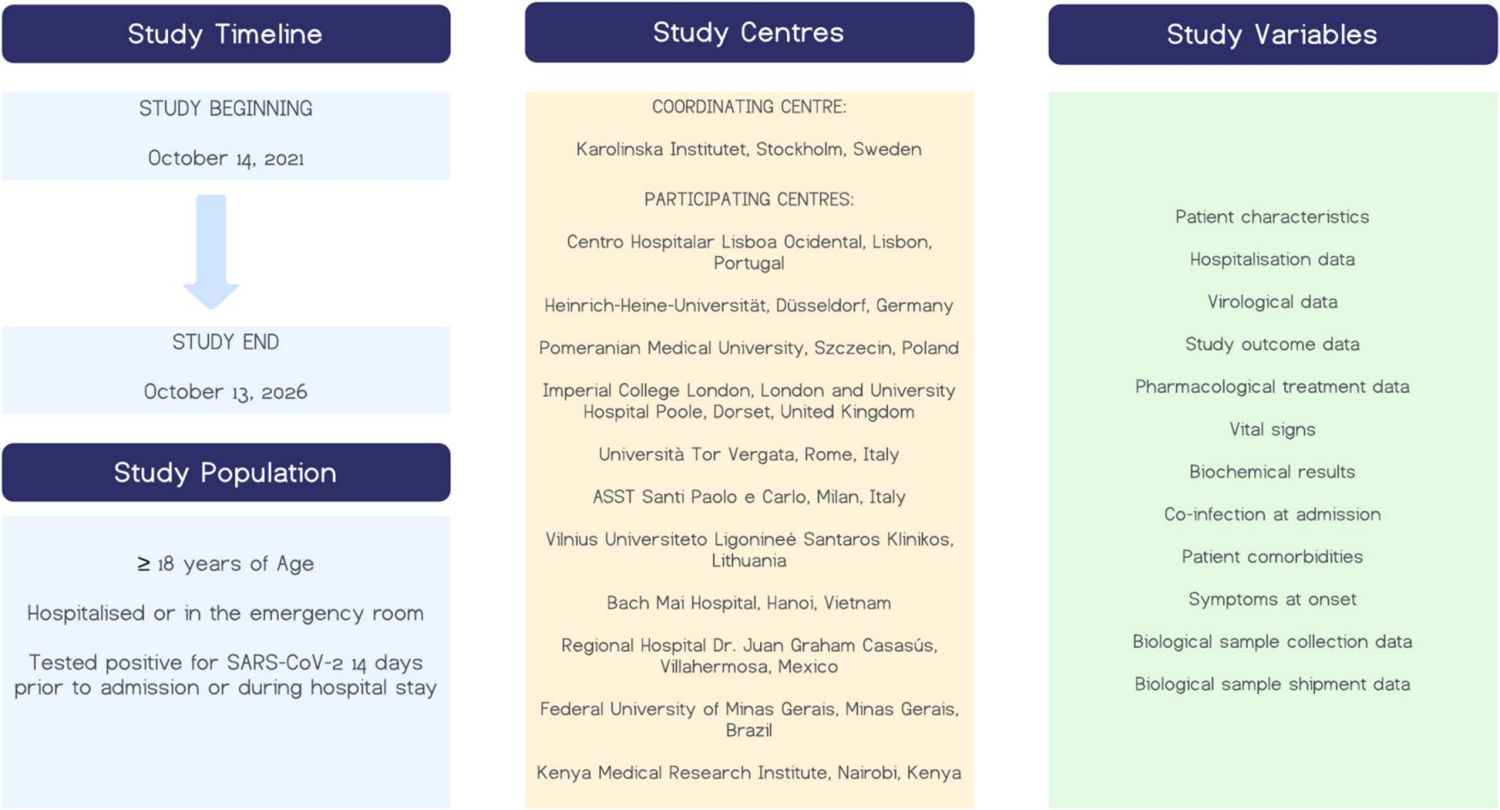
Study timeline, study population, study centres, and study variables

Observational multicentre combined retrospective and prospective study (Fig. 1).

### Study timeline

The study commenced on 14th October 2021 and is expected to conclude on 13th October 2026 (Fig. 1).

### Study population

The study population includes adult patients (≥ 18 years) who are hospitalised or who have died in the emergency department and have tested positive for SARS-CoV-2 by polymerase chain reaction (PCR) or by an antigen test within 14 days prior to admission or during hospitalisation. The study aims to include both first and recurrent episodes of COVID-19. All study subjects are required to sign informed consent when required by ethical approval. Patients who do not meet these inclusion criteria will be excluded from the study.

The anticipated sample size for the study is 35,000, which is expected to increase depending on the course of the COVID-19 pandemic. Due to the recruitment strategy of the study, the exact sample size cannot be predetermined as it is dependent on the number of patients admitted to participating centres. This approach will provide a diverse cohort of patients from various geographical regions, with a range of demographic and clinical characteristics, thus establishing a robust foundation for the study's research objectives (Fig. 1).

### Study centres

The coordinating centre will be Karolinska Institutet, Stockholm, Sweden. Other participating centres are: Centro Hospitalar Lisboa Ocidental, Lisbon, Portugal; Heinrich-Heine-Universität Düsseldorf, Düsseldorf, Germany; ASST Santi Paolo e Carlo, Milan, Italy; Imperial College London, London, United Kingdom and University Hospital Dorset, Poole, United Kingdom; Vilnius University Hospital Santaros Klinikos, Vilnius, Lithuania; University of Rome Tor Vergata (UNITOV), Rome, Italy; Bach Mai Hospital, Hanoi, Vietnam; Kenya Medical Research Institute (KEMRI), Nairobi, Kenya; Regional Hospital Dr. Juan Graham Casasús, Villahermosa, Mexico; Federal University of Minas Gerais, Minas Gerais, Brazil; Pomeranian Medical University, Szczecin, Poland (Fig. 1).

### Variables definition and data collection

The variables in this study have been meticulously defined, considering the study objectives and the availability and structure of data at the participating centres. This process involved iterative harmonisation. Adhering to the principles of the General Data Protection Regulation (GDPR), the principle of data minimisation was adopted to facilitate data collection. The collected data encompass various aspects, including demographics, SARS-CoV-2 virological variables, COVID-19-specific treatments, clinical outcomes, vital signs, laboratory parameters, and co-morbidities. These variables are categorised into mandatory and non-mandatory, with the mandatory ones being crucial for addressing the study objectives. Data collection strictly adheres to the study protocol, the most recent version of the Declaration of Helsinki, the International Council for Harmonisation of Technical Requirements for Pharmaceuticals for Human Use – Good Clinical Practice (ICH-GCP) or ISO EN 14155, the GDPR, as well as all applicable national legal and regulatory requirements.

To ensure robust data management, a dedicated data management suite has been developed, employing a security by design architecture. The suite is hosted on a secure Google business environment. It enables manual data entry into case report forms and facilitates bulk data upload via secure file transfer. This flexibility accommodates the diverse data infrastructures at the participating centres, where data is collected either in bulk from electronic health record-based databases or through manual review of medical charts. After initial data pre-processing and curation steps, the collected data are stored in a central relational database. This database includes several tables such as: patient information, hospitalisation data, SARS-CoV-2 virological data, study outcome, COVID-19 pharmacological treatment data, vital signs, clinical chemistry data, admission co-infection information, diagnoses/comorbidities, symptoms, biological samples and shipment information. In addition to clinical data, biological materials such as serum/plasma, peripheral blood mononuclear cells, nasopharyngeal blood viral isolates, and/or viral sequences are collected from selected patients for further analysis in WP2.

Whole blood samples are drawn and stored centrifugated and separated into plasma and/or serum. Whole blood is drawn in 2 × 4.5 mL tubes for analysis. In a subset of patients, peripheral blood mononuclear cells (PBMCs) are separated by Ficoll-Isopaque for assessment of cellular immunity against SARS-CoV-2. Serum and/or plasma are used for neutralisation assays and for assessment of SARS-CoV-2 diagnostics. The collection of biological samples is not mandatory and is performed only on a subset of patients.

In summary, the biological materials – where taken—includes:

- Plasma or serum samples for titration of neutralising antibodies.
- PBMCs for assessment of any pre-existing or vaccine-induced cellular-mediated immunity response to the different variants.
- Virus isolates from clinical specimens to enrich the repository of SARS-CoV-2 variants. Isolation procedures will be run as available at individual laboratories.
- When available, SARS-CoV-2 variant information is collected. In addition, full-length viral genomic RNA sequencing is run by the next-generation sequencing (NGS) platform available at the individual laboratories for selected patients.

Specific regulations exist regarding the international shipment of biological samples derived from study subjects. It is the responsibility of the investigator (assisted by the courier service and the coordinating centre) to ensure that all study samples for international transport are appropriately handled, packed, and shipped.

Plasma samples are stored in a repository/biobank at the site or shipped to the designated laboratory of the consortium, at -80° Celsius, and will be destroyed no later than December 31, 2045. All samples stored will be analysed and destroyed in accordance with current legal and ethical requirements.

Pseudo-anonymised patient data as well as viral variant data are collected in a secure centralised server at EuResist Network where all the cleaning and refactoring procedures will be run. Cleansed, harmonised data will then be stored in a relational database in the same centralised server. MariaDB RDBMS is being used both for the collection of data via the eCRF and for the storage of the cleansed data (Fig. 1).

### Data analysis

Data analysis for this study is tailored to each specific study objective. All final analyses will be conducted by an assigned study statistician or through common statistical packages disseminated to each study site. Descriptive data of the study cohort will be presented with medians and interquartile ranges for continuous variables and number and percentages for categorical variables. This will be presented in an overall, pooled format, as well as stratified by study sites and study time-period.

The main exposure of interest, SARS-CoV-2 variants, will be studied using both the currently described genotypes of main interest and a subset of whole genome sequencing data. Depending on local and temporal variations in circulating variants and the percentage of hospitalised patients with variant classification performed, the variant might be extrapolated based on the current variant distribution.

The primary outcome, in-hospital mortality, will be studied using Cox proportional hazards models and Fine and Gray models, with the underlying baseline hazard function based on the date of admission or the date of the first positive test if tested positive later during hospitalisation. Patients with community-onset and hospital-onset COVID-19 will be analysed both pooled and separately. Competing risks analyses will be used to examine ICU admission, length of hospitalisation, and length of ICU stay, with in-hospital death as a competing event.

Covariate adjustment will be used to control for potential confounding variables, including age, sex, ethnicity, co-morbidities in accordance with risk factors for severe COVID-19 according to the Centre for Disease Control and Prevention (CDC), SARS-CoV-2 vaccination status, and geographical region. Both complete case analyses and analyses based on multiple imputation by chained equations imputed or inverse probability weighted datasets will be performed to account for missing data.

Given the evolving nature of COVID-19, covariates as well as other variables and definitions may change over the course of the pandemic. The effect of various interventions performed during hospitalisation on the study objectives will be evaluated using causal inference methods, including inverse propensity weighting and doubly robust methods, to account for potential treatment biases and violations of positivity assumptions.

Finally, machine learning (ML) methods will be used to analyse data, including unsupervised ML methods for heterogeneous multi-view data and supervised ML methods that correct for phylogenetic relationships and other potential biases. These state-of-the-art methods will provide a comprehensive and nuanced understanding of the data and its implications for the study objectives.

### Study sponsor

The WP3 hospitalised cohort study is investigator-initiated and coordinated by EuResist, a European Economic Interest Grouping based in Rome, Italy. The study is financed by European Union (EU) Horizon Europe (Grant Number 101046016) and overseen by the EuCARE project’s Scientific Management Board. Participating clinics will receive reimbursement for enrolment and follow-up data collection. However, no compensation will be provided to study participants.

### Data dissemination

The study aims to publish its findings in peer-reviewed journals and present them at national and international conferences, with a preference for open access journals. Authorship will be determined based on academic standards and conventions, acknowledging all investigators and contributors in compliance with recognised standards. The publications and presentations will be listed on the EuCARE webpage.

## Discussion

The EuCARE project is a multifaceted initiative that aims to provide an advanced response to the ongoing COVID-19 epidemics. Among the various cohorts within the EuCARE project, the EuCARE-HOSPITALISED study stands out for its focus on assessing the relationship between different circulating variants and the risk of in-hospital mortality. It defines a wide range of variables, including epidemiological and clinical factors, as well as advanced laboratory features, to analyse the progression, management, and outcomes of COVID-19 disease. One of the key strengths of the EuCARE-HOSPITALISED study is its large sample size, which makes it one of the largest cohorts studied to date. This provides increased statistical power to detect even subtle relationships between different circulating variants and in-hospital mortality. Additionally, the study's wide range of variables allows for a comprehensive analysis of the progression, management, and outcomes of COVID-19 disease, incorporating epidemiological and clinical factors, as well as advanced laboratory features. Furthermore, the collection of biologic samples from hospitalised patients in the EuCARE-HOSPITALISED study offers opportunities for future research on important aspects that have not yet been investigated. This is particularly relevant as COVID-19 is a relatively new disease, and much is still unknown about its pathophysiology, clinical presentation, and long-term outcomes. Another notable aspect of the EuCARE-HOSPITALISED study is its collaboration with machine-learning experts. By leveraging the power of artificial intelligence (AI) to integrate both patient and viral data, researchers can develop advanced predictive models that may help identify patients at higher risk of in-hospital mortality and guide clinical decision-making. The long duration of the EuCARE-HOSPITALISED study is also noteworthy. Covering different phases and waves of COVID-19, along with differences in viral characteristics, clinical management, and vaccination status, it provides a dynamic perspective on the disease and epidemics. This is crucial considering the rapidly evolving nature of COVID-19, where factors influencing disease progression and outcomes may change over time. Moreover, it's important to highlight that the EuCARE-HOSPITALISED study benefits from the insights provided by a distinguished external scientific and stakeholder advisory board, as well as an ethics advisory board. Finally, The EuCARE-HOSPITALISED study aims to investigate the clinical outcome and differences in mortality among COVID-19 patients based on their response to antivirals and the booster vaccination dose received.

The study has some limitations that are common to observational studies. These include the potential for unmeasured confounding and confounding by indication. Additionally, missing data for some participants may introduce bias and reduce the statistical power of the analysis. Another potential source of bias is the use of variant genotyping or whole genome sequencing, which may have been conducted selectively for different individuals or at different time periods and study sites. This variability may introduce bias due to differences in indications for variant characterisations at different time periods and study sites.

Overall, the EuCARE-HOSPITALISED study represents a comprehensive and valuable effort to evaluate the association between different circulating variants and in-hospital mortality in COVID-19 patients. The findings of this study may have important implications for patient care, public health policy, and future research endeavours.

## Data Availability

The datasets that are used and/or analysed during the current study are available from the corresponding author on reasonable request.
